# Rare Late Pleistocene-early Holocene human mandibles from the Niah Caves (Sarawak, Borneo)

**DOI:** 10.1371/journal.pone.0196633

**Published:** 2018-06-06

**Authors:** Darren Curnoe, Ipoi Datan, Jian-xin Zhao, Charles Leh Moi Ung, Maxime Aubert, Mohammed S. Sauffi, Goh Hsiao Mei, Raynold Mendoza, Paul S. C. Taçon

**Affiliations:** 1 ARC Centre of Excellence for Australian Biodiversity and Heritage, Palaeontology, Geobiology and Earth Archives Research Centre, School of Biological, Earth and Environmental Sciences, University of New South Wales, Sydney, New South Wales, Australia; 2 Sarawak Museum Department, Jalan Barak, Kuching, Sarawak, Malaysia; 3 School of Earth and Environmental Sciences, University of Queensland, Brisbane, Queensland, Australia; 4 Griffith Centre for Social and Cultural Research, Australian Research Centre for Human Evolution, Griffith University, Gold Coast Campus, Queensland, Australia; 5 Centre for Global Archaeological Research, Universiti Sains Malaysia, Penang, Malaysia; 6 PERAHU, Griffith Centre for Social and Cultural Research, School of Humanities, Languages and Social Science, Griffith University, Gold Coast campus, Queensland, Australia; Max Planck Institute for the Science of Human History, GERMANY

## Abstract

The skeletal remains of Late Pleistocene-early Holocene humans are exceptionally rare in island Southeast Asia. As a result, the identity and physical adaptations of the early inhabitants of the region are poorly known. One archaeological locality that has historically been important for understanding the peopling of island Southeast Asia is the Niah Caves in the northeast of Borneo. Here we present the results of direct Uranium-series dating and the first published descriptions of three partial human mandibles from the West Mouth of the Niah Caves recovered during excavations by the Harrissons in 1957. One of them (mandible E/B1 100") is somewhat younger than the ‘Deep Skull’ with a best dating estimate of c30-28 ka (at 2σ), while the other two mandibles (D/N5 42–48" and E/W 33 24–36") are dated to a minimum of c11.0–10.5 ka (at 2σ) and c10.0–9.0 ka (at 2σ). Jaw E/B1 100" is unusually small and robust compared with other Late Pleistocene mandibles suggesting that it may have been ontogenetically altered through masticatory strain under a model of phenotypic plasticity. Possible dietary causes could include the consumption of tough or dried meats or palm plants, behaviours which have been documented previously in the archaeological record of the Niah Caves. Our work suggests a long history back to before the LGM of economic strategies involving the exploitation of raw plant foods or perhaps dried and stored meat resources. This offers new insights into the economic strategies of Late Pleistocene-early Holocene hunter-gatherers living in, or adjacent to, tropical rainforests.

## Introduction

Island (or ‘maritime’) Southeast Asia (iSEA) spans a geographic area of approximately 3 million km^2^ and comprises close to 20,000 islands. While researchers have investigated the prehistory of the region for over 130 years [1,2], large gaps remain in our understanding of the Palaeolithic as well as the culture and history of more recent hunter-gatherer populations. From a global standpoint, the Late Pleistocene dispersal of early Anatomically Modern Humans (AMH) across iSEA is widely regarded to be a key issue for understanding the evolution of the human species [3]. In recent decades, archaeologists have largely focused on three major aspects of this problem: 1) the timing and migration routes that may have been followed during the settlement of iSEA [4,5]; 2) the cultural adaptations and subsistence strategies employed by early modern humans across diverse environments [6–8]; and 3) the identity and physical adaptations of the people/populations involved in the Late Pleistocene peopling of the region as well as its later hunting and gathering inhabitants [9,10]. The present article centers on the final of these three issues.

Despite the long history of archaeological research in iSEA, the remains of Late Pleistocene AMH have been found at only four localities. These comprise partial skulls and postcranial elements from Wadjak in Indonesia discovered in 1888 (Wadjak-1) by B.D. van Rietschoten and in 1890 (Wadjak-2) by Eugene Dubois [11]; and most recently estimated to be c35 ka [12]. Also in Indonesia, at Lida Ajer Cave, Dubois found two human teeth in 1887 and 1890, and these have recently been proposed to be c73-63 ka [5]. Further north, in the Philippines, James Fox [13,14] recovered a sample of human remains at Tabon Cave during excavations undertaken in the 1960s, which were extrapolated from ^14^C dating of charcoal to be 22,000–24,000 B.P. [12]. More recently, excavations at Tabon Cave in 2000 recovered more skeletal elements, and these have been dated with the direct Uranium-series (U-series) method to c17 (frontal fragment), c31 ka (mandible) and c47 ka (tibia) [14]. We note also that human remains have been also recovered from Callao Cave in the Philippines including a metatarsal of uncertain taxonomic affinity dated with the U-series method to a minimum age of c67 ka [15] and an undescribed tooth dated to c50 ka [12].

**Fig 1:**
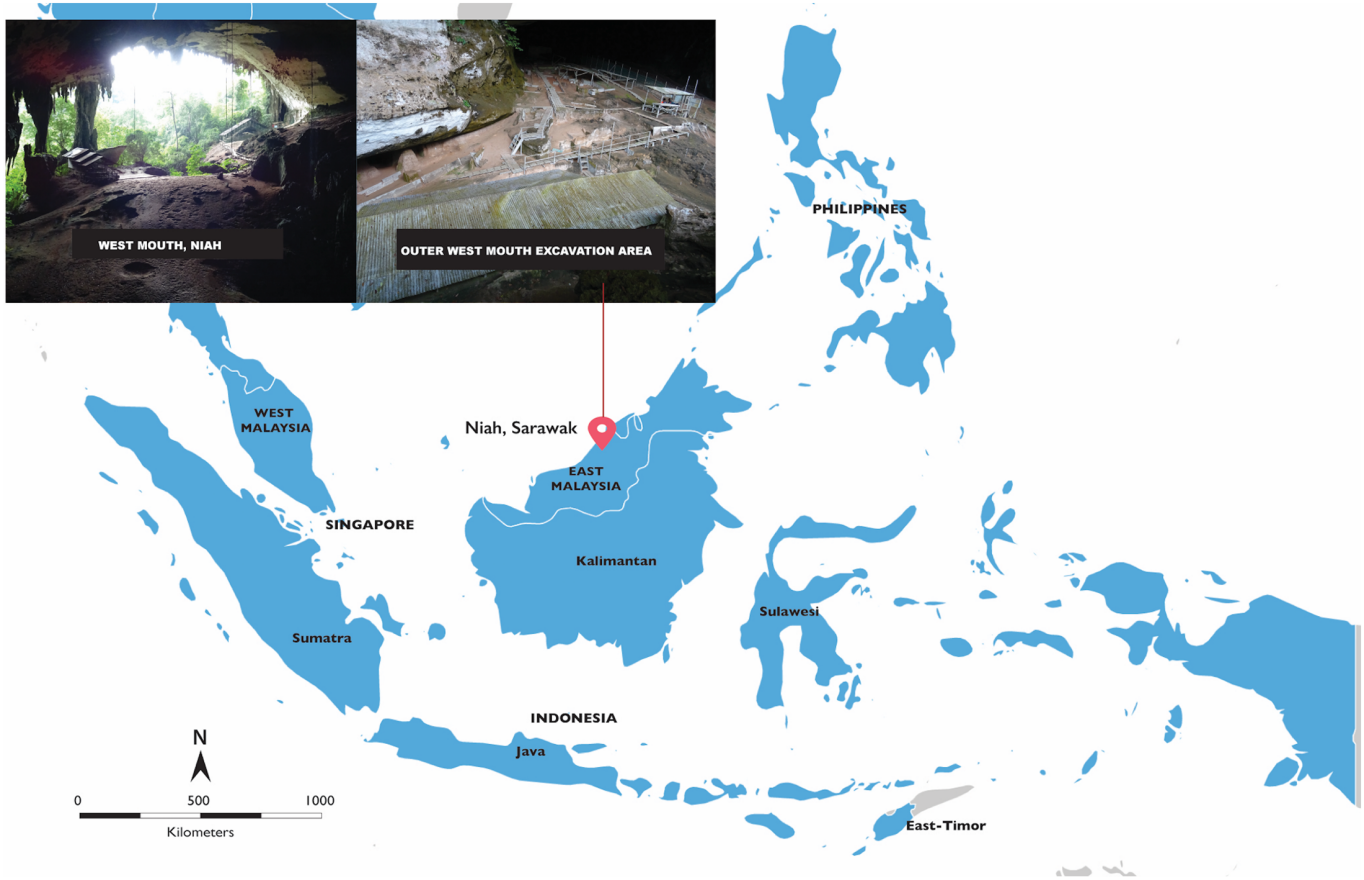
The West Mouth of the Niah Caves and its location within island Southeast Asia (Credits: photographs D. Curnoe; map adobe stock under an extended license).

In the centre of iSEA lies the Niah Great Cave complex (Batu Niah) on the island of Borneo in East Malaysia (Sarawak) (Fig 1) where the ‘Deep Skull’ was found in Kuala Besar (West Mouth) by the Tom and Barbara Harrisson in 1958 [16–18]. These remains, comprising a partial cranium with maxillary dentition, partial femur, tibia and talus [10,19,20], have most recently been estimated with direct U-series dating on two fragments of skull bone to 35.2±2.6 ka (error weighted average) [21], and through Bayesian modeling combining AMS ^14^C of charcoal and direct U-series dating of human bone c39-30 ka (at 94.5 per cent probability) [22].

The Niah Great Cave is a spectacular karst system located about 80 km southwest of Miri in the northeast of Sarawak (Fig 1). It was formed through solution and rapid tectonic uplift of the Miocene age Mount Subis limestone massif [23]. From 1947 onwards, Tom Harrisson began archaeological survey and excavations at the Niah Caves, initially with Michael Tweedie and Hugh Gibb, and later with Barbara Harrisson. During research lasting from 1954 to 1967, they excavated in several caves in the Niah Great Cave, their work yielding a vast archaeological and palaeontological collection spanning the Late Pleistocene through to late Holocene [10,17,18,20,24–28].

In the West Mouth, the Harrisons also excavated more than 250 isolated human bones and partial skeletons designating many of them ‘burials’ [17]. Late Pleistocene deposits are extensive and cover a horizontal area of roughly 700 m^2^. They were exposed during excavations in the ‘Hell Trench,’ where the Deep Skull was recovered, and adjoining deposits, as well as in Areas A and B (as designated by the Niah Caves Project) of the Harrisson archaeological zone [29–31]. The base of these deposits has a minimum age of c50 ka [29–31] making the West Mouth of the Niah Caves broadly contemporaneous with other early AMH localities in iSEA. A further 11 Iron Age inhumations were exposed during excavations led by Majid in 1977 [32], while Barker and co-workers investigated an additional 12 burials during fieldwork from 2000–2003 [33]. In total, 262 burials have been formally recognised from the West Mouth, the majority of them being associated with a Neolithic cemetery dating c4-2 ka [33]. However, a collection of fragmentary human remains recovered by the Harrissons in the 1950s, some of which were archived until recently among the fauna from the West Mouth [30], have remained unstudied. Based on their excavation grid coordinates and recent reconstructions of the chronostratigraphic sequences of the West Mouth [29–31] they might be Late Pleistocene-early Holocene in age.

**Fig 2:**
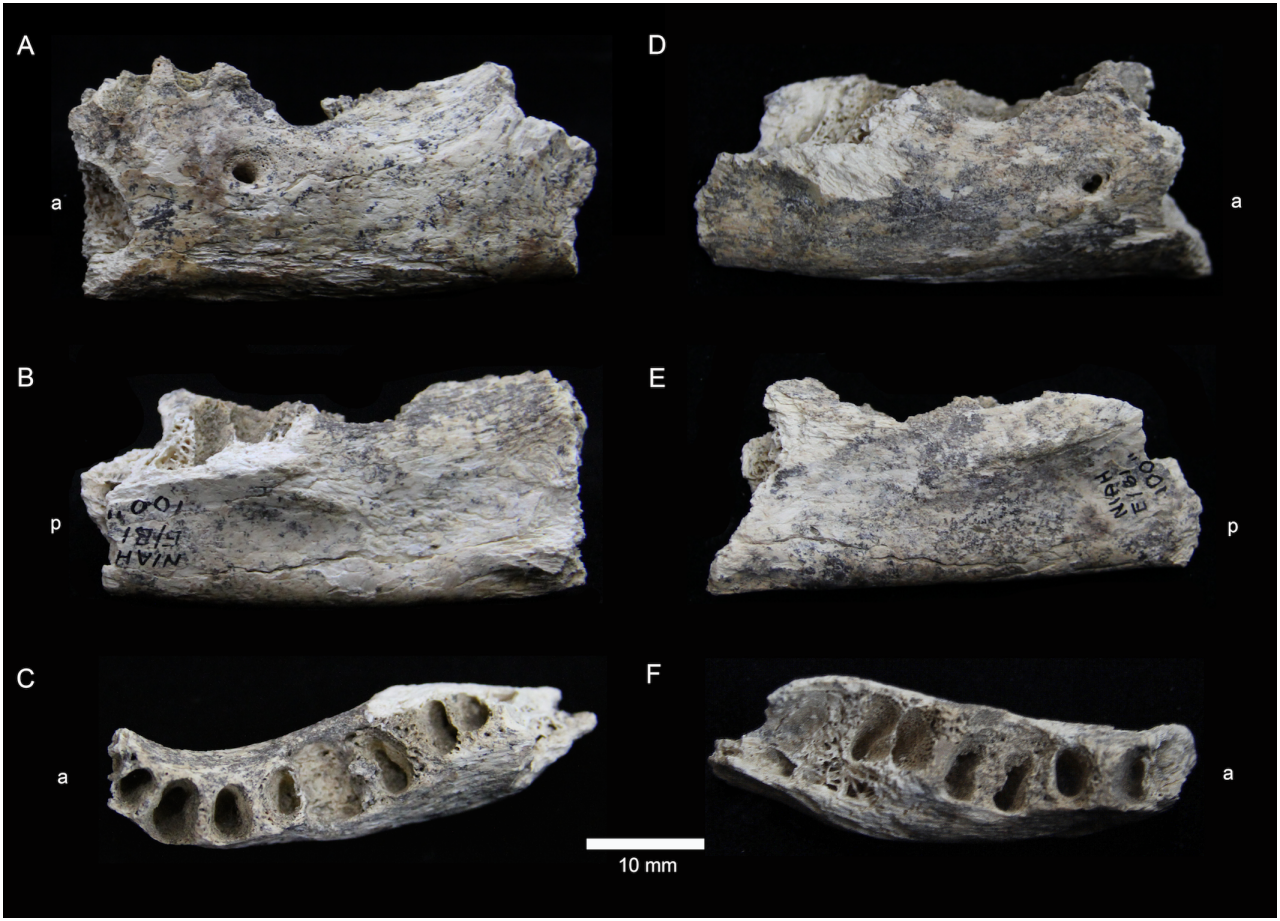
Human mandible E/B1 100" from the West Mouth of the Niah Caves. A. Left fragment, lateral view. B. Left fragment, medial view. C. Left fragment, superior view. D. Right fragment, lateral view. E. Right fragment, medial view. F. Right, superior view. a = anterior, p = posterior.

**Fig 3:**
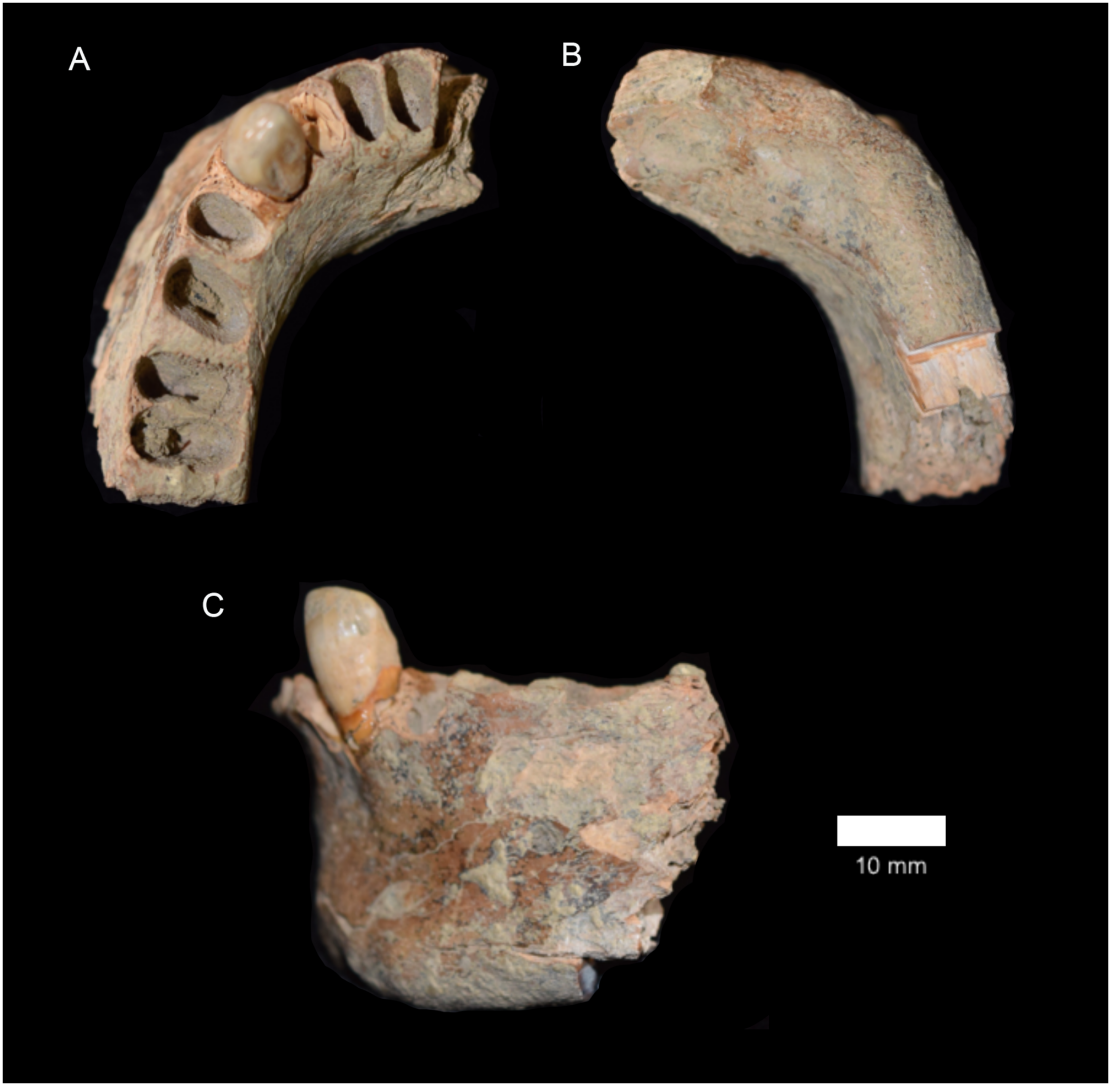
Human mandible D/N5 42–48" from the West Mouth of the Niah Caves. A. Superior view. B. Inferior view. C. Left lateral view.

It is within this context that we present the results of direct U-series dating and the first published descriptions of three partial human mandibles (Figs 2 and 3, S1 and S2 Tables, S1 Fig) from Area A and the Area A/B intersection the West Mouth of the Niah Caves recovered during excavations by the Harrissons in 1957.

## Materials and methods

### Ethics statement

All of the human remains studied in the present research are stored at the Sarawak Museum Department, Kuching, Sarawak (Malaysia). Permission to collect mandibles from the Neolithic cemetery at Niah Caves (Burials B121, B183, B189 and B206; see S3 Table) and to undertake all research on mandibles E/B1 100", D/N5 42–48" and E/W33 24–36" was provided by the Director of the Sarawak Museum Department and the State Planning Unit of the Sarawak Government (in accordance with Research Permit JKM/SPU/608-8/2/2/2 Vol.2, issued to Darren Curnoe as project leader).

### Uranium-series analyses

Conventional U-Th dating was conducted using a Nu Plasma multi-collector inductively-coupled plasma mass spectrometer (MC-ICP-MS) in the Radiogenic Isotope Facility (RIF) at the School of Earth and Environmental Sciences, The University of Queensland (UQ) following chemical treatment procedures and MC-ICP-MS analytical protocols described elsewhere [34,35]. Fossil samples weighing 1.5–2.5 mg were spiked with a mixed ^229^Th-^233^U tracer and then completely dissolved in concentrated HNO3. After digestion, each sample was treated with H_2_O_2_ to decompose trace amounts of organic matter and to facilitate complete sample-tracer homogenisation. U and Th were separated using conventional anion-exchange column chemistry using Bio-Rad AG 1-X8 resin. After stripping off the matrix from the column using double-distilled 7N HNO as eluent, 3 ml of a 2% HNO3 solution mixed with trace amount of HF was used to elute both U and Th into a 3.5-ml pre-cleaned test tube, ready for MC-ICP-MS analyses, without the need for further drying down and re-mixing. After column chemistry, the U-Th mixed solution was injected into the MC-ICP-MS through a DSN-100 desolvation nebuliser system with an uptake rate of around 0.07 ml per minute. U-Th isotopic ratio measurement was performed on the MC-ICP-MS using a detector configuration to allow simultaneous measurements of both U and Th isotopes [34,36]. The ^230^Th/^238^U and ^234^U/^238^U activity ratios of the samples were calculated using the decay constants given in [34]. The non-radiogenic ^230^Th was corrected using an assumed bulk-Earth atomic ^230^Th/^232^Th ratio of 4.4±2.2×10^−6^. U-Th ages were calculated using the *Isoplot* Program [37].

For laser ablation U-series analyses a cut was made perpendicular to the bone surface using a rotatory tool equipped with a thin (100 μm wide) diamond saw blade. The cut sample was mounted into an aluminium cup, aligning the cross-sectioned surface with the outer rim of the sample holder to position the samples on the focal plane of the laser in the sampling cell. Sequential laser spot analyses were undertaken along two parallel tracks, starting from the interior of the cross-sectioned bone. The analyses were made at regular spacing (typically 2–3 mm) along each track, using a laser spot size of 265 μm and a 5 Hz pulse rate. The samples were initially cleaned for 5s and ablation pits were measured for 50s. U-series ages were calculated using the *Isoplot* program [37]. Closed system ages were calculated for each spot analysis and an age estimate (and the two-sigma ± errors) was calculated for each parallel track. The two sigma ± error is given as a combination of standard error of the mean and average relative error. Laser ablation U-series dating was undertaken using the MC-ICP-MS system at the Australian National University’s Research School of Earth Sciences. The details of laser ablation U-series analysis of skeletal remains were recently summarised in [12].

### Morphological and metrical comparisons

A sample of late Holocene (Neolithic) mandibles from *in situ* exposed burials was collected by us from the West Mouth of Niah Caves in 2017. These were collected from the surface of graves originally excavated by the Harrisons and left exposed in the cave. As we did not undertake additional excavations to recover these bones, the size of the sample is necessarily small. Morphological observations on all of the Niah Cave mandibles were made using standard methods [38,39]. Mandibular body areas were calculated by treating the body section as an ellipse, and using the formula Height x Width x π /4 [40]. Data from additional samples were compiled from the relevant literature [11,41–53] (see S3 Table).

## Results

### Uranium-series dating

Nitrogen testing of two bone samples from E/B1 100" determined that collagen was insufficient for the application of ^14^C analysis. The same problem has been encountered by researchers working previously on bone samples from Niah Caves including the Deep Skull [54,55]. Therefore, we conducted U-series analysis to directly estimate the geological age of the mandibles; an approach that has been successfully applied previously to the Deep Skull [21,22,26,30].

U-series analysis provides insights into when U has migrated into a bone. This may happen a short or long time after the burial of a skeletal element, while later U-overprints may also exist which can be difficult to distinguish. As such, apparent U-series results from bones are generally regarded to be minimum age estimates. Unlike in corals and speleothems, U-Th dating of bone is based on the premise that U is taken up from the environment by bone apatite that scavenges U, but excludes Th, during diagenesis. Fresh bone contains little or no U, so U uptake can occur only post-mortem. Yet, it can be difficult, even impossible, to determine by how much U-series results underestimate the true age of a bone sample. In ideal cases, U uptake may have reached saturation during the early stages of fossilization (early uptake mode). In this case, the U-Th date would approximate the depositional age of the fossil [56]. However, in most cases U uptake modes are likely to have been more complex [57] so that the U-Th dates of the fossil are theoretically variable, but invariably younger than the estimated age of a fossil [58]. Because of the complex U-uptake history even in different parts of the same bone, apparent U-Th dates may vary across a single sample. All of them should, however, represent minimum ages of deposition, with the oldest being closest to the true burial age of the bone. In some cases, the preferential loss of U relative to Th owing to subsequent leaching of soluble U from bones may result in a ^230^Th/^238^U ratio that is apparently too high, leading to age overestimation [56]. Such variation cannot easily be detected using conventional (solution) U-series analysis. However, U leaching can be recognized on the basis of combined U concentration and U isotope profiling across samples using the laser ablation method.

The results of U-series dating are summarised in Table 1 (S4 and S5 Tables, S2 Fig). Conventional U-series dating provided a minimum age for E/B1 100" of 25.5±0.2 ka, or c26-25 ka at 2σ, establishing that it derives from the Late Pleistocene (Table 1). The mean minimum age of E/B1 100" using laser ablation analysis is 21.7±0.5 ka, given as the mean value of all individual spot analyses (Table 1). Additionally, the youngest age for the fragment was 18.8±1.0 ka (Spot-12) but the oldest was 28.9±1.0 ka (Spot-27), indicating that U-uptake by this bone could have been a protracted process lasting at least 10 ka. As the maximum U-series age should be closest to the true age of the bone E/B1 100" is probably best considered to be c30-28 ka (at 2σ), or slightly older than the solution minimum age for the mandible.

**Table 1:** Summary results of direct U-series dating of human mandibles from the West Mouth of the Niah Caves^a^.

	U (ppm)	^232^Th (ppb)	Age (ka)
E/B1 100"			
Solution	3.01±0.01	5.61±0.01	25.5±0.2
Laser ablation			
Mean	2.21±0.01	3.94±0.01	21.7±0.5
Maximum	2.12±0.01	6.09±0.01	28.9±1.0
D/N5 42–48" (solution)	4.34±0.00	2.54±0.02	10.6±0.1
E/W 33 24–66" (solution)	1.32±0.00	16.99±0.02	9.4±0.3

^a^Errors are at 2σ. Further details are provided in S4 and S5 Tables.

**Fig 4:**
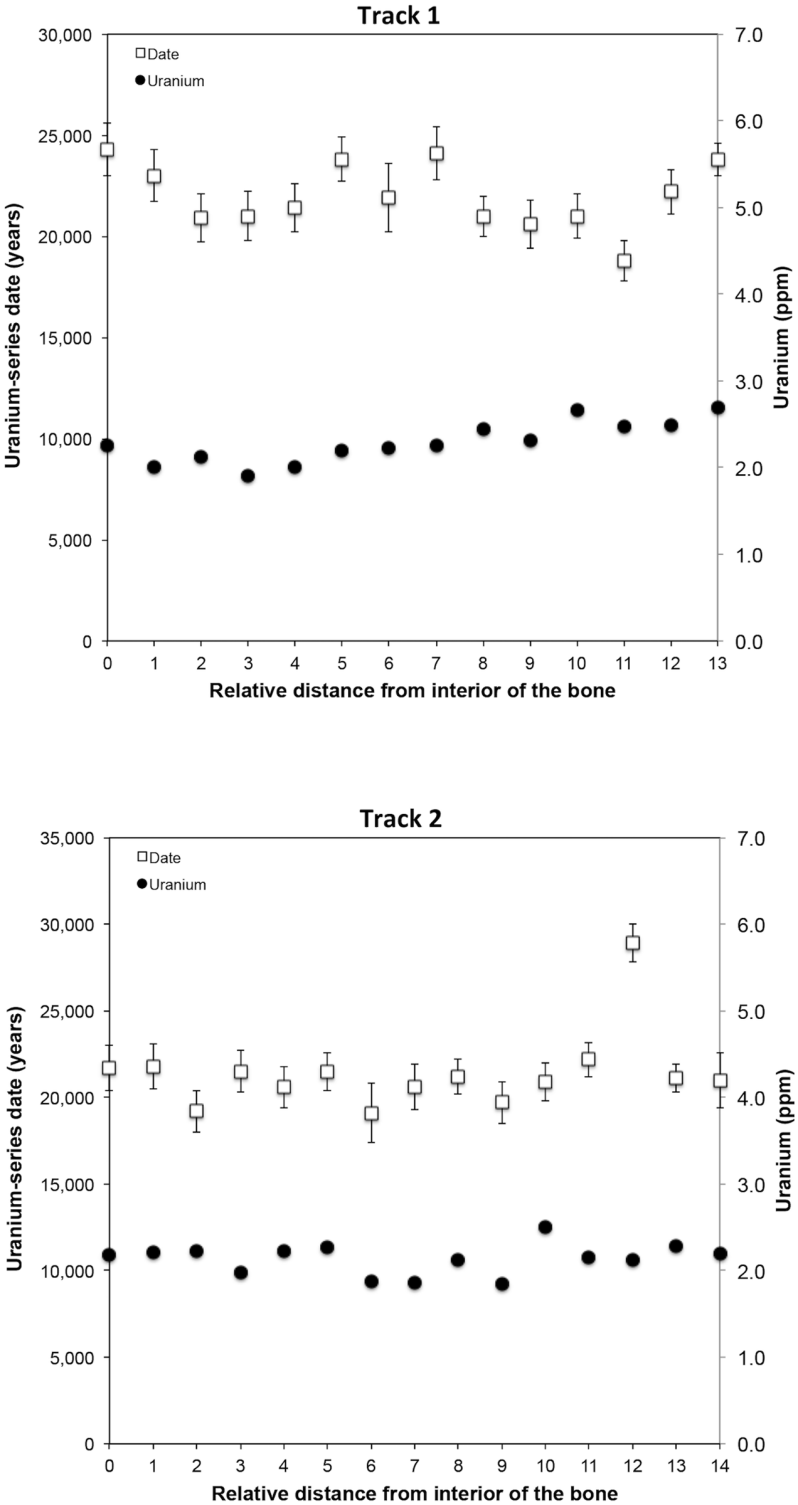
Uranium and U-series date profile for tracks 1 and 2 of human mandible E/B1 100" bone fragment from the West Mouth of the Niah Caves. Errors on the individual dates are 2σ.

The uranium profile of a bone fragment taken from the medial surface of E/B1 100" determined from laser ablation analysis along two tracks was found to be uniform (Fig 4, S2 Fig), its shape indicating that the bone had reached equilibrium with the uranium in the groundwater. While laser ablation U-series age calculations may be compromised if the U-concentrations are below about 0.5 ppm, they were well above this threshold for E/B1 100". The age and uranium profiles of Track 1 and Track 2 indicate slightly different uptake behavior (Fig 4). Track 1 documents a relatively even increase in uranium concentration accompanying distance from the inner part of the bone, while Track 2 is more variable and lacks any such trend. The presence of detrital ^232^Th, which may derive from either sediment contained within pores or diffusion from external sediment, can also compromise U-series dating results. When the elemental U/Th ratios drops below 300 the resulting U-series result may be influenced by detrital ^230^Th. This was the case for only two spots in Track 2 (Spot-16 and Spot-29) where the U/Th ratios were 265 and 233 respectively (S5 Table). The bone sample from E/B1 100" lacked any obvious signs of U leaching.

Mandible D/N5 42–48" was dated only with solution U-series and determined to be 10.6±0.1 ka, or c11.0–10.5 ka at 2σ. Jaw fragment E/W 33 24–66" was dated with solution U-series dating to 9.4±0.3 ka, or c10.0–9.0 ka at 2σ. Both mandibles derive from the early Holocene (Table 1, S4 Table).

### Preservation and morphology

Mandible E/B1 100" comprises two partial (left and right) fragments of an adult mandibular body lacking completely the dentition (Fig 2). The left has a total length of 58.8 mm and the right is 60 mm long. The middle section of the left is largely complete from about the canine alveolus to the M_2_ alveolus, but there is evidence for superficial damage during excavation. The LM_1_ mesial root alveolus has been modified by pathology notable as a well circumscribed area of reactive bony infill possibly associated with an apical cyst. There seems to have been slight expansion (mediolaterally) of the body as a result, but it is confined to this region (mesial M_1_ alveoli). The right body is preserved from just behind P_1_ posteriorly, P_2_-M_1_ alveoli being intact, and preserving the mesial alveolar wall and septum between the mesial and distal roots and partial mesial root alveoli of M_3_.

The body of E/B1 100" is relatively ruggedly built indicating well-developed musculature associated with the oral handling and processing of food and accompanying broadening or buttressing of the body. There is a deep sublingual fossa accompanied above by a prominent alveolar shelf along the course of the molars. In addition, the mylohyoid line is clearly demarcated and runs parallel to the alveolar margin, fading out by about mid-M_1_. On the inner margin of the remaining symphysis the superior and inferior transverse tori are observed to be enlarged with the superior torus forming a small shelf. The digastric fossae are incised and face posteriorly, while the sublingual fossae are broad and relatively deep structures.

The mental foramen (MF) sits within a broader hollowing of bone and is surrounded by a bony rim (left and right). There is observable pitting around the superior section of the rim of the foramen on both sides suggestive of hypervascularization. The MF is accompanied by a small accessory foramen which is located within the anterior margin of its rim (both sides). The MF is located beneath the P_2_ in E/B1 100" (left and right), which is the most common state for mainland and island Southeast Asian and Northeast Asian mandibles (83/100%), although, it is less frequent in European mandibles (60%), the small sample of Neolithic mandibles from the West Mouth (33%) and Sahul mandibles (10%) (Table 2). E/B1 100" also lacks a retromolar space, which is the most common condition seen among Late Pleistocene/early Holocene (LPH) humans from all regions except island Southeast Asia and Sahul, where is it present in all mandibles (Table 2). Although the medial section of the symphysis is missing externally from E/B1 100", in inferior view the rim of the body is observed to flatten out anteriorly as it arches on approach to form a mentum osseum (chin), confirming its status as an AMH.

**Table 2:** Comparison of discrete traits for human mandibles from the West Mouth of the Niah Caves^a^^,^^b^.

Sample	Mental foramen % mesial of P_2_/M_1_	Retromolar space % absent	Chin Size Rank %-4 and 5
E/B1 100"	P_2_	Absent	-
D/N5 42–48"	P_2_/M_1_	-	Rank 3
NC-NEO	33% (3)	100% (4)	-
mSEA-LPH	83% (6)	50% (4)	100% (1)
iSEA-LPH	100% (2)	0% (2)	-
NEA-LPH	89% (9)	79% (8)	100% (7)
SAH-LPH	10% (10)	0 (8)	-
EUP-LPH	60% (12)	79% (34)	98.4% (38)

^a^NC-NEO, Niah Caves Neolithic; mSEA-LPH, mainland Southeast Asia Late Pleistocene/early Holocene humans; iSEA-LPH, island Southeast Asia Late Pleistocene/early Holocene humans; NEA-LPH, Northeast Asia Late Pleistocene/early Holocene humans; SAH-LPH, Sahul Late Pleistocene/early Holocene humans; EUP-LPH, Europe Late Pleistocene/early Holocene humans.

^b^(n).

Mandible D/N5 42–48" (Fig 3) comprises the complete left side of a mandibular body from I_1_ to M_1_ alveoli. The left minimally worn I_2_ and C crowns were preserved *in situ* but have subsequently dislodged during storage. The body is complete anteroposteriorly from the symphysis to beneath the mid-alveolar septum separating the medial and distal M_1_ root alveoli, where after it is missing. On the right side, the I_1_ alveolar is complete but the body is broken from just lateral to the incomplete I_2_ alveolus.

The modestly sized and circular mental foramen sits beneath P_2_/M_1_ and opens in the superior direction. This location is most frequent in Niah Caves Neolithic (67%) and Sahul mandibles (90%), common in European LPH mandibles (40%), but far less common among mainland and island Southeast Asian (0/17%) and Northeast Asian (11%) individuals (Table 2). Internally, there is a prominent (thick) bar of bone behind the symphysis, resulting from an enlarged superior transverse torus, and medially, a thickened inferior torus. Laterally, on the left side, there is a deep subalveolar fossa, but the genial spines are unremarkable. The well-developed (i.e. deep, anteroposteriorly and mediolalerally wide) digastric fossa faces somewhat posteriorly. The sublingual fossa on the left side (right missing) is a broad and moderately deep structure. The alveolar ridge is absent both internally and externally.

All of the elements of a modern human chin are in place in mandible D/N5 42–48": the mental tubercle is a clearly demarcated but not especially large structure, being about ‘Rank 3’ according to the scale of [59], which is comparatively small and rarely observed among LPH human mandibles (Table 2). There is a clear mental trigone with a well-developed lateral (mental) tubercle; the left mental fossa is preserved and is evident upon palpation, but quite shallow; superiorly a sharp (but thin) vertical keel is present and runs from the apex of the mental trigone to the alveolar margin above. The base of the body is a prominent and rounded thickening, and it thins medially on either side compared to the adjacent body.

The final mandible fragment we examined is the very incomplete E/W33 24–36" (S1 Fig). It comprises a small section of the right body from the bony septum between P_4_-M_1_, including part of the medial septal wall, to the septum between M_2_-M_3_. The *in situ* pulp cavities and roots of M_1_ and M_2_ are largely intact but missing the dental crowns, which are broken away from below the cervical margin. The lateral body is preserved in its approximate superior most half, while medially it is preserves less than its superior third, from mid-P_2_ lateral alveolar wall posteriorly to just posterior to the M_1_-M_2_ septum. The preserved fragment provides very little morphological data, and none that are useful for assessing its affinities.

### Metrical comparisons

Mandible E/B1 100" presents as a small and robust mandibular body (Table 3, S1 Table). At the level of the MF, its height (average: 22.0 mm) is 2.5σ below the pooled LPH sample mean (30.8±3.5 mm, n30); none of the known mainland or island Southeast Asian or Northeast Asian mandibles (26.0–36.8 mm, n12), nor the mandibles from a small sample of Neolithic burials we examined from the West Mouth of the Niah Caves are this short. Body width at the level of the MF is moderately thick in E/B1 100" (12.8 mm) being only 0.2σ below the pooled LPH sample mean (13.2±2.0 mm, n26), and well within the range of all LPH samples (Table 3). It is, however, well below the range of the sampled Neolithic mandibles from the West Mouth (13.7–16.0 mm). Body height at the MF in D/N5 42–48" (29.6 mm) is unremarkable and sits well within the range of all LPH samples (Table 3, S3 Table). Its body is quite wide/thick (14.2 mm) and most closely resembles the Niah Cave Neolithic, mainland Southeast Asian and Sahul mandibles (Table 3); it is, however, only 0.5σ below the pooled LPH sample mean.

**Table 3:** Body dimensions and shape index at location of mental foramen (MF) of human mandibles from the West Mouth of Niah Caves^a^.

Sample	MF height (mm)	MF width (mm)	MF Area (mm^2^)	MF shape (%)
E/B1 100" (L/R)	22.5/21.5	12.8/12.8	226.2/216.1	56.9/59.5
D/N5 42–48" (L)	29.6	14.2	420.3	48.0
NC-NEO	29.6,30.8,36.0	13.7,14.2,16.0	330.1,331.4,452.4	44.4,44.5,50.0
mSEA-LPH	29.6±2.4 (4)	13.6±2.2 (4)	317.5±68.7 (4)	45.6±5.2 (4)
iSEA-LPH	30.5 (1)	12.0 (1)	287.5 (1)	39.3 (1)
NEA-LPH	30.3±3.0 (8)	12.4±0.8 (5)	305.6±35.0 (5)	40.1±5.1 (5)
SAH-LPH	36.0,36.0	12.5,14.5,19.0	353.4,410.0	34.7,40.3
EUP-LPH	29.5±7.6 (10)	12.3±1.7 (11)	281.3±105.2 (10)	37.7±12.1 (10)

^a^μ±σ (n). “Area” calculated using the formula for an ellipse (see Materials and methods); “Shape”, Width/Height x 100. See footnote to Table 2 for sample abbreviations.

Body area at the MF in E/B1 100" (average: 221.15 mm^2^) is also very small, being 1.8σ below the pooled LPH sample mean (315.1±53.8 mm^2^, n22), and well below the ranges (minima) of Southeast/Northeast Asian (224.6–388.1 mm^2^, n10) and Neolithic mandibles from the West Mouth (330.1–452.4 mm^2^, n3). However, the body area of the larger D/N5 42–48" mandible (330.1 mm^2^) lies well within the range of all LPH samples (Table 3). Lastly, body shape index at the level of the MF in large in both specimens: E/B1 100" (average 58.2%), being outside of the range of all samples, and D/N5 42–48" (48%), sitting above the maximum value for LPH mandibles; with mandible E/B1 100" being 2.6σ above the pooled LPH sample mean (41.0±6.7%, n22) (Table 3). Incidentally, the marked thickness of the body is observed also at the level of M_1_/M_2_ where E/B1 100" is 3.4σ below the pooled LPH sample mean (30.9±3.4%, n22; data from S3 Table pooled and weighted using statistics μ ± σ from [50]).

Table 4 compares the size and shape of the symphysis of D/N5 42–48". Its symphysis height (31.6 mm), thickness (16.6 mm), calculated area (412.0 mm^2^) and shape index (52.5%) are unremarkable, their values sitting within 1σ of the pooled LPH sample mean, and within 1σ of sample averages where it can be compared (Table 4).

**Table 4:** Symphysis dimensions and shape index of Niah Caves of human mandible D/N5 42–48" from the West Mouth of Niah Caves^a^.

Sample	Symphysis height (mm)	Symphysis width (mm)	Symphysis Area (mm^2^)	Symphysis shape (%)
D/N5 42–48"	31.6	16.6	412.0	52.5
NC-NEO	29.5 (1)	17.4 (1)	403.1 (1)	59.0 (1)
mSEA-LPH	31.6±1.8 (5)	14.0 (1)	351.9 (1)	43.8 (1)
iSEA-LPH	30.5,40.2	17.8 (1)	562.0 (1)	44.3 (1)
NEA-LPH	31.5±2.7 (6)	-	-	-
SAH-LPH	38.3±3.4 (35)	16.0±1.7 (39)	480.5±71.4 (34)	41.3±5.0 (34)
EUP-LPH	32.2±3.4 (15)	15.9±1.6 (8)	400.2±67.9 (8)	50.1±6.6 (8)

^a^μ±σ (n). “Area” calculated using the formula for an ellipse (see Materials and methods); “Shape”, Width/Height x 100; See footnote to Table 2 for sample abbreviations.

Finally, in Table 5 we present and compare dental crown width (labiolingual diameter) for D/N5 42–48". Overall, they are, comparatively speaking, rather unremarkable. The I^2^ is slightly narrowed (6.5 mm), but identical to average values for recent Negrito and Malay samples, as well being within the range of all other comparative samples (Table 5). The canine is somewhat enlarged in width (8.4 mm); but its value sits within 1σ of all sample averages except Nerito (1.5σ above the average) and mainland Southeast Asian LPH (-1.3σ below the sample average) (Table 5).

**Table 5:** Dental crown width for human mandible D/N5 42–48" from the West Mouth of the Niah Caves^a^.

Sample	I_2_ (mm)	C (mm)
D/N5 42–48"	6.5	8.4
Dayak	6.4±0.7 (7)	7.9±0.6 (13)
Negritos	6.5±0.4 (15)	7.7±0.5 (9)
Malay	6.5±0.5 (4)	8.0±0.6 (6)
mSEA-LPH	6.8±0.3 (24)^b^	9.3±0.7 (27)^b^
iSEA-LPH	6.8 (1)	7.7 (1)
NEA-LPH	6.3,6.9,7.1	8.0±2.9 (9)^c^
SAH-LPH	6.8±0.5 (20)	8.8±0.6 (29)
EUP-LPH	6.9±0.5 (28)	8.6±0.7 (26)

^a^μ±σ (n). See footnote to Table 2 for sample abbreviations.

^b^Pooled and weighted μ±σ of individual teeth from the literature and samples from [45].

^c^Pooled and weighted μ±σ of samples from [50].

## Discussion

Of the three mandibles from the West Mouth of the Niah Caves we have analysed here, E/B1 100" stands out owing to its secure dating to the Late Pleistocene and unusual morphology. Given that U-series dating of bone provides only a minimum age, the conventional date for the specimen of c26-25 ka (at 2σ) should be viewed as a minimum age only. However, as the maximum laser ablation date should probably be closest to the true age of the specimen, the age for E/B1 100" is more likely to be within the range c30-28 ka (at 2σ). This is somewhat younger (by up to 9 ka) than the estimated age for the oldest human remains from the site (the Deep Skull) as determined through Bayesian modeling combining AMS ^14^C and U-series of c39-30 ka (at 94.5 per cent probability) [22].

The scarcity of Pleistocene AMH remains across iSEA brings into focus the importance of the Niah Caves jaws, especially E/B1 100". It is similar in age to human mandibles from Wadjak (Indonesia) and Tabon Cave (Philippines), and along with the Deep Skull, they together provide direct evidence of penecontemporary human populations living across a broad segment of the region prior to the Last Glacial Maximum. The Wadjak and Tabon mandibles are clearly from very large bodied individuals [11,60], however, they fall well short of E/B1 100" in terms of absolute and relative body thickness and, therefore, robusticity. Its excessive body robusticity might best be considered to have resulted from masticatory loading that occurred during ontogeny and arising from dietary and/or non-alimentary behaviours involving the jaws and teeth.

The morphology of the mandible preferentially over the remaining skull reflects masticatory pressure rather than neutral population history [61]. There are a number of well-documented examples of the effects of masticatory loading on the mandibular morphology among hunter-gatherers. For example, comparisons of the mandibles of the Alaskan Tigara people, who largely consumed a diet of dried meat, with those of South Dakota Arikara, who practiced a varied economic strategy combining horticulture and seasonal hunting, have shown major differences in morphology resulting from greater growth in the buccolingual (width) dimension of the body [62]. Moreover, experimental research on mammals fed varying diets has shown that varying food softness can result in substantial (~10%) reductions in the growth of the mandible and other regions of the viscerocranium that experience high strain [63].

Other morphological indicators of hypertrophy of the muscles of the oral cavity seen in E/B1 100" might include deep sublingual fossae and molar shelving, thickening of the superior and inferior transverse tori and hollowing of the digastric fossae. These would also reflect masticatory strain under a model of phenotypic plasticity, perhaps involving a diet that included hard and brittle foods with minimal preparation or even the use of the teeth as tools. In the case of the Niah remains, possible dietary causes might include the consumption of tough or dried meats or underground storage organs [64,65]. The zooarchaeological record from some parts of Niah Cave such as Terminal Pleistocene deposits in Lobang Hangus shows extensive exploitation of animal foods and interestingly little evidence for hearths or cooking [66]. Moreover, studies of starch granules from the West Mouth sediments have also suggested that palm was consumed by the human occupants of the cave and would have been eaten raw [66]. Either or both of these behaviours, if practiced extensively through the sub-adult years, might result in a robust mandibular body.

## Conclusions

We have provided for the first time the results of direct-dating and morphological investigations of three human mandibles found more than 60 years ago by the Harrissons in the archaeological zone of the West Mouth of Niah Caves. The rarity of Late Pleistocene-early Holocene human remains from across island Southeast Asia underscores their importance in helping to fill a major gap in understanding about the early hunting and gathering populations of the region. Our work also shows that Niah Caves might document a long history stretching back to before the LGM of economic strategies involving the exploitation of raw plant foods or perhaps even dried and stored meat resources. This potentially also offers some new insights into the economic adaptations of Late Pleistocene-early Holocene hunter-gatherers living in, or adjacent to, tropical rainforests, the economic difficulties of which are well known [67].

## Supporting information

S1 Table Body dimensions (mm) for mandible E/B1 100". (DOCX)

S2 Table Body dimensions (mm) for mandible D/N5 42–48". (DOCX)

S3 Table Raw data from Neolithic mandibles from the West Mouth of the Niah Caves and published mandibles. (DOCX)

S4 Table U-Th isotope data from solution U-series analysis for three human mandibles from the West Mouth of the Niah Caves. (DOCX)

S5 Table Results from laser ablation U-series analysis of mandible E/B1 100". (DOCX)

S1 Fig Mandible E/W 33 24–66" from the West Mouth of the Niah Caves. (DOCX)

S2 Fig Fragment of bone from medial surface of E/B1 100" showing the two laser tracks from laser ablation U-series analyses. (DOCX)
