# Supplementary material for: Rare Late Pleistocene-early Holocene human mandibles from the Niah Caves (Sarawak, Borneo)
Source: PLoS One. 2018 Jun 6;13(6):e0196633. doi: 10.1371/journal.pone.0196633 (PMC5991356; doi:10.1371/journal.pone.0196633)
Supplement: S1 Table — (DOCX) [file pone.0196633.s001.docx]

**S1 Table. Body dimensions (mm) for mandible E/B1 100".^a^**

|  | Height |  | Width |  |
| --- | --- | --- | --- | --- |
|  | L | R | L | R |
| I_2_-C | (21.0) | - | 11.6 | - |
| Canine | - | - | 11.6 | - |
| C/P_1_ | 23.5 | - | 11.9 | - |
| Mental foramen | 22.5 | 21.5 | 12.8 | 12.8 |
| M_1_/M_2_ | 18.8 | 19.5 | 16.7 | (16.4) |
| M_2_ | 19.5 | - | - | (16.2) |

^a^Values in parenthesis are estimates owing to damaged bone.
