# Supplementary material for: Rare Late Pleistocene-early Holocene human mandibles from the Niah Caves (Sarawak, Borneo)
Source: PLoS One. 2018 Jun 6;13(6):e0196633. doi: 10.1371/journal.pone.0196633 (PMC5991356; doi:10.1371/journal.pone.0196633)
Supplement: S2 Table — (DOCX) [file pone.0196633.s002.docx]

**S2 Table. Body dimensions (mm) for mandible D/N5 42-48".**

|  | Height | Width |  |
| --- | --- | --- | --- |
| Symphysis | 31.6 | 16.6 | |
| Mental foramen | 29.6 | 14.2 | |
| P_1_/P_2_ | 31.0 | 15.7 | |
| M_1_ | 28.7 | 14.4 | |
