# Supplementary material for: Rare Late Pleistocene-early Holocene human mandibles from the Niah Caves (Sarawak, Borneo)
Source: PLoS One. 2018 Jun 6;13(6):e0196633. doi: 10.1371/journal.pone.0196633 (PMC5991356; doi:10.1371/journal.pone.0196633)
Supplement: S3 Table — (DOCX) [file pone.0196633.s003.docx]

**S3 Table. Raw data from Neolithic mandibles from the West Mouth of the Niah Caves and from published mandibles.**

|  | Mental  For. Location | Mental For. H (mm) | Mental For. T  (mm) | Shape Index  (%) | Area  (mm^2^) | Symphysis H  (mm) | Symphysis  T  (mm) | Shape  Index  (%) | Area  (mm^2^) | M^1^/M^2^ H  (mm) | M^1^/M^2^ T  (mm) | Shape  Index  (%) | Area  (mm^2^) |
| --- | --- | --- | --- | --- | --- | --- | --- | --- | --- | --- | --- | --- | --- |
| Niah B183 | P2 | 30.8 | 13.7 | 44.5 | 331.4 | 29.5 | 17.4 | 59.0 | 403.1 | - | - | - | - |
| Niah B206 | M1 | 36 | 16 | 44.4 | 452.4 | - | - | - | - | - | - | - | - |
| Tianyuan 1 | P2 | 29.7 | 12.1 | 40.7 | 282.2 | 27.5 | - | - | - | 29.6 | 11.7 | 39.5 | 272.0 |
| Minatogawa 1 | P2 | 30.9 | - | - | - | 30.5 | - | - | - | - | - | - | - |
| Minatogawa A | P1/P2 | 27.9 | - | - | - |  | - | - | - | - | - | - | - |
| Minatogawa B | M1 | 28.7 | 13.4 | 46.7 | 302.0 | 31.1 | - | - | - | - | - | - | - |
| Minatogawa C | P2 | 27.1 | - | - | - | - | - | - | - | - | - | - | - |
| Zkoukoudian UC101 | P2 | 36.8 | 12.6 | 34.2 | 364.2 | - | - | - | - | 33.9 | 14.2 | 41.9 | 377.5 |
| Zkoukoudian UC104 | P2 | 30 | 12.9 | 43.0 | 303.9 | - | - | - | - | 26.5 | 15.0 | 56.6 | 312.2 |
| Zkoukoudian UC108 | P2 | 31.3 | 11.2 | 35.8 | 275.3 | - | - | - | - | 30.0 | 12.2 | 40.7 | 272.0 |
| Gua Gunung | P2 | 31 | 14 | 45.2 | 340.9 | 34 | - | - | - | - | - | - | - |
| Moh Kiew | P2 | 31 | 13 | 41.9 | 316.5 | 32 | - | - | - | - | - | - | - |
| Hang Cho | P2 | 26 | 11 | 42.3 | 224.6 | 31 | - | - | - | - | - | - | - |
| Mai Da Nuoc | P2 | - | - | - | - | 32 | 14 | 43.8 | 351.9 | - | - | - | - |
| Mai Da Dieu | P2 | - | - | - | - | 29 | - | - | - | 34 | 19 | 55.9 | 507.4 |
| Wadjak 1 |  |  |  |  |  |  |  |  |  | 37 | 21 | 56.8 | 610.3 |
| Wadjak 2 | P2 | - | - | - | - | 40.2 | 17.8 | 44.3 | 562.0 | - | - | - | - |
| Liang Lembudu | P2 | 30.5 | 12 | 39.3 | 287.5 | 30.5 | - | - | - | - | - | - | - |
| Tam Pa Ling | P2/M1 | 30.5 | 16.2 | 53.1 | 388.1 | - | - | - | - | - | 18.3 | - | - |
| Tabon PXIIT436-Sq19 | - |  |  |  |  |  |  |  |  | - | 15 | - | - |
| Tabon un-numbered | - |  |  |  |  |  |  |  |  | - | 21 | - | - |
| Watinglo | P2/M1 | - | 19 |  |  | 30.5 | - | - | - | - | - | - | - |
| Kow Swamp 1 | P2/M1 | 36 | 12.5 | 34.7 | 353.4 | 38 | 15 | 39.5 | 447.7 | - | - | - | - |
| Kow Swamp 5 | P2/M1 | 36 | 14.5 | 40.3 | 410.0 | 38 | 15 | 39.5 | 447.7 | - | - | - | - |
| Kow Swamp 7 | P2/M1 | - | - | - | - | 36 | 19 | 52.8 | 537.2 | - | - | - | - |
| Kow Swamp 9 | - | - | - | - | - | 39 | - | - | - | - | - | - | - |
| Kow Swamp 14 | - | - | - | - | - | 39 | 16 | 41.0 | 490.1 | - | - | - | - |
| Kow Swamp 53 | P2/M1 | - | - | - | - | - | - | - | - | - | - | - | - |
| Kow Swamp 55 | P2/M1 | - | - | - | - | - | - | - | - | - | - | - | - |
| Kow Swamp 65 | P2/M1 | - | - | - | - | - | - | - | - | - | - | - | - |
| Kow Swamp 70 | P2/M1 | - | - | - | - | - | - | - | - | - | - | - | - |
| Cossack | P2 | - | - | - | - | 31 | 16 | 51.6 | 389.6 | - | - | - | - |
| Mossgiel | - | - | - | - | - | 29 | 21.0 | 72.4 | 478.3 | - | - | - | - |
| Mungo 3 | - | - | - | - | - | 32 | 14.5 | 45.3 | 364.4 | - | - | - | - |
| Dolni Vestonice 3 | P2 | 24.2 | 12.7 | 52.5 | 241.4 | 25.5 | 15 | 58.8 | 300.4 | - | - | - | - |
| Dolni Vestonice 13 | P2 | 29 | 12.3 | 42.4 | 280.2 | 31.3 | 17 | 54.3 | 417.9 | - | - | - | - |
| Dolni Vestonice 14 | P2 | 28.5 | 10 | 35.1 | 223.8 | 30.3 | 12.5 | 41.3 | 297.5 | - | - | - | - |
| Dolni Vestonice 15 | P2 | 27.9 | 12 | 43.0 | 263.0 | 30.7 | 15.8 | 51.5 | 381.0 | - | - | - | - |
| Dolni Vestonice 16 | P2 | 37 | 12.8 | 34.6 | 372.0 | 35.5 | 16.9 | 47.6 | 471.2 | - | - | - | - |
| Pavlov 1 | P1 | 36.5 | 11.3 | 31.0 | 323.9 | 35.5 | 15.9 | 44.8 | 443.3 | - | - | - | - |
| Muierii 1 | P2/M1 | - | 11.6 | - | - | - | - | - | - | - | - | - | - |
| Sunghir | - | 36 | 11 | 30.6 | 311.0 | - | - | - | - | - | - | - | - |
| Oase 1 | - | 33.5 | 11.9 | 35.5 | 313.1 | 34.5 | - | - | - | - | - | - | - |
| l'Abri Pataud | - | 30 | 15 | 50.0 | 353.4 | 29 | - | - | - | - | - | - | - |
| Gough's Cave | - | 32 | 16 | 50.0 | 402.1 | 31 | 18 | 58.1 | 438.3 | - | - | - | - |
| Predmost 1 | - | - | - | - | - | 33 | - | - | - | - | - | - | - |
| Predmost III | - | - | - | - | - | 39 | - | - | - | - | - | - | - |
| Predmost IX | - | - | - | - | - | 31 | - | - | - | - | - | - | - |
| Predmost X | - | - | - | - | - | 30 | - | - | - | - | - | - | - |
| Obercassel | - | - | - | - | **-** | 30 | - | - | - | - | - | - | - |
| Mladec 52 | - | - | - | - | **-** | 36 | 16 | 44.4 | 452.4 | - | - | - | - |
