# Supplementary material for: Rare Late Pleistocene-early Holocene human mandibles from the Niah Caves (Sarawak, Borneo)
Source: PLoS One. 2018 Jun 6;13(6):e0196633. doi: 10.1371/journal.pone.0196633 (PMC5991356; doi:10.1371/journal.pone.0196633)
Supplement: S4 Table — (DOCX) [file pone.0196633.s004.docx]

**S4 Table. U-Th isotope data from solution U-series analysis for three human mandibles from the West Mouth of the Niah Caves.**

| Mandible | U  (ppm) | ±2σ | ^232^Th  (ppb) | ±2σ | (^230^Th/  ^232^Th) | ±2σ | (^230^Th/  ^238^U) | ±2σ | (^234^U/  ^238^U) | ±2σ | Uncorr. Age  (ka) | ±2σ | Corr.  Age  (ka) | ±2σ | Corr. Initial (^234^U/  ^238^U) | ±2σ |
| --- | --- | --- | --- | --- | --- | --- | --- | --- | --- | --- | --- | --- | --- | --- | --- | --- |
| E/B1 100" | 3.0056 | 0.0009 | 5.612 | 0.012 | 369.0 | 3.1 | 0.2271 | 0.0018 | 1.0872 | 0.0016 | 25.50 | 0.23 | 25.45 | 0.24 | 1.0938 | 0.0018 |
| D/N5 42-48" | 4.3383 | 0.0023 | 2.535 | 0.017 | 512.5 | 5.1 | 0.0987 | 0.0007 | 1.0652 | 0.0022 | 10.61 | 0.09 | 10.59 | 0.09 | 1.0672 | 0.0022 |
| E/W 33 24-66" | 1.3176 | 0.0006 | 16.994 | 0.018 | 21.01 | 0.4 | 0.0893 | 0.0017 | 1.0477 | 0.0020 | 9.72 | 0.20 | 9.36 | 0.27 | 1.0491 | 0.0021 |

Note: Ratios listed in the table refer to activity ratios normalized to the corresponding ratios measured for the secular-equilibrium HU-1 standard. ^230^Th ages are calculated using Isoplot/Ex 3.75 [36], using decay constants of Cheng et al. [33]. Non-radiogenic ^230^Th correction was applied assuming non-radiogenic ^230^Th/^232^Th atomic ratio = 4.4±2.2 x 10^-6^ (bulk-earth value), and ^238^U, ^234^U, ^232^Th and ^230^Th are in secular equilibrium. uncorr. and corr. denote uncorrected and corrected.
