# Supplementary material for: Rare Late Pleistocene-early Holocene human mandibles from the Niah Caves (Sarawak, Borneo)
Source: PLoS One. 2018 Jun 6;13(6):e0196633. doi: 10.1371/journal.pone.0196633 (PMC5991356; doi:10.1371/journal.pone.0196633)
Supplement: S5 Table — (DOCX) [file pone.0196633.s005.docx]

**S5 Table. Results from laser ablation U-series analysis of mandible E/B1 100".**

|  |  | **U (ppm)** | **Th (ppb)** | **U/Th** | **230Th/238U** | **230Th/238U** | **234U/238U** | **234U/238** | **CS Age (ka)** | **CS Age error (ka)** |
| --- | --- | --- | --- | --- | --- | --- | --- | --- | --- | --- |
|  |  |  |  |  |  | **error** | **error** | **error** |  |  |
| Track-1 | Spot-1 | 2.26 | 5.47 | 414 | 0.2192 | 0.0101 | 1.0961 | 0.0095 | 24.3 | 1.3 |
| Track-1 | Spot-2 | 2.01 | 3.18 | 630 | 0.211 | 0.0101 | 1.1055 | 0.0102 | 23 | 1.3 |
| Track-1 | Spot-3 | 2.13 | 3.53 | 604 | 0.1895 | 0.0101 | 1.0859 | 0.0069 | 20.9 | 1.2 |
| Track-1 | Spot-4 | 1.91 | 1.81 | 1055 | 0.1916 | 0.01 | 1.0917 | 0.0102 | 21 | 1.2 |
| Track-1 | Spot-5 | 2.01 | 3.48 | 578 | 0.197 | 0.0096 | 1.1016 | 0.0099 | 21.4 | 1.2 |
| Track-1 | Spot-6 | 2.2 | 1.63 | 1353 | 0.2117 | 0.0087 | 1.0785 | 0.0096 | 23.8 | 1.1 |
| Track-1 | Spot-7 | 2.23 | 3.56 | 626 | 0.2024 | 0.0137 | 1.1098 | 0.0091 | 21.9 | 1.7 |
| Track-1 | Spot-8 | 2.26 | 5.32 | 425 | 0.2176 | 0.0102 | 1.0952 | 0.0084 | 24.1 | 1.3 |
| Track-1 | Spot-9 | 2.45 | 2.68 | 914 | 0.1948 | 0.0085 | 1.1077 | 0.0083 | 21 | 1 |
| Track-1 | Spot-10 | 2.31 | 3.24 | 711 | 0.1905 | 0.0099 | 1.1016 | 0.0089 | 20.6 | 1.2 |
| Track-1 | Spot-11 | 2.66 | 4.51 | 590 | 0.1931 | 0.0092 | 1.099 | 0.0081 | 21 | 1.1 |
| Track-1 | Spot-12 | 2.47 | 4.19 | 589 | 0.1744 | 0.0082 | 1.0966 | 0.0096 | 18.8 | 1 |
| Track-1 | Spot-13 | 2.49 | 2.65 | 938 | 0.2001 | 0.0086 | 1.0842 | 0.0078 | 22.2 | 1.1 |
| Track-1 | Spot-14 | 2.69 | 1.95 | 1381 | 0.2194 | 0.0067 | 1.1168 | 0.0069 | 23.8 | 0.8 |
| Track-2 | Spot-15 | 2.18 | 4.35 | 502 | 0.2002 | 0.0135 | 1.1075 | 0.0093 | 21.7 | 1.6 |
| Track-2 | Spot-16 | 2.21 | 8.32 | 265 | 0.199 | 0.0102 | 1.095 | 0.0091 | 21.8 | 1.3 |
| Track-2 | Spot-17 | 2.22 | 4.99 | 445 | 0.178 | 0.0083 | 1.0979 | 0.0101 | 19.2 | 1 |
| Track-2 | Spot-18 | 1.98 | 4.78 | 413 | 0.197 | 0.0099 | 1.0971 | 0.0111 | 21.5 | 1.2 |
| Track-2 | Spot-19 | 2.23 | 3.09 | 723 | 0.1888 | 0.0082 | 1.0938 | 0.0098 | 20.6 | 1 |
| Track-2 | Spot-20 | 2.27 | 7.35 | 309 | 0.1966 | 0.0097 | 1.0973 | 0.0079 | 21.5 | 1.2 |
| Track-2 | Spot-21 | 1.88 | 1.54 | 1219 | 0.1807 | 0.0092 | 1.1199 | 0.0089 | 19.1 | 1.1 |
| Track-2 | Spot-22 | 1.86 | 2.69 | 692 | 0.1912 | 0.0096 | 1.1057 | 0.01 | 20.6 | 1.2 |
| Track-2 | Spot-23 | 2.12 | 6.35 | 334 | 0.1971 | 0.0103 | 1.1106 | 0.0097 | 21.2 | 1.2 |
| Track-2 | Spot-24 | 1.85 | 1.39 | 1332 | 0.1825 | 0.0084 | 1.1003 | 0.0102 | 19.7 | 1 |
| Track-2 | Spot-25 | 2.51 | 1.37 | 1832 | 0.1938 | 0.0093 | 1.1058 | 0.0088 | 20.9 | 1.1 |
| Track-2 | Spot-26 | 2.15 | 2.66 | 807 | 0.2019 | 0.0109 | 1.0932 | 0.0091 | 22.2 | 1.4 |
| Track-2 | Spot-27 | 2.12 | 6.09 | 347 | 0.2593 | 0.0077 | 1.1099 | 0.0079 | 28.9 | 1 |
| Track-2 | Spot-28 | 2.28 | 2.71 | 841 | 0.1926 | 0.0068 | 1.0925 | 0.0083 | 21.1 | 0.8 |
| Track-2 | Spot-29 | 2.2 | 9.44 | 233 | 0.1922 | 0.008 | 1.0969 | 0.0113 | 21 | 1 |
