# Supplementary figures and images for: Rare Late Pleistocene-early Holocene human mandibles from the Niah Caves (Sarawak, Borneo)
Source: PLoS One. 2018 Jun 6;13(6):e0196633. doi: 10.1371/journal.pone.0196633 (PMC5991356; doi:10.1371/journal.pone.0196633)

**
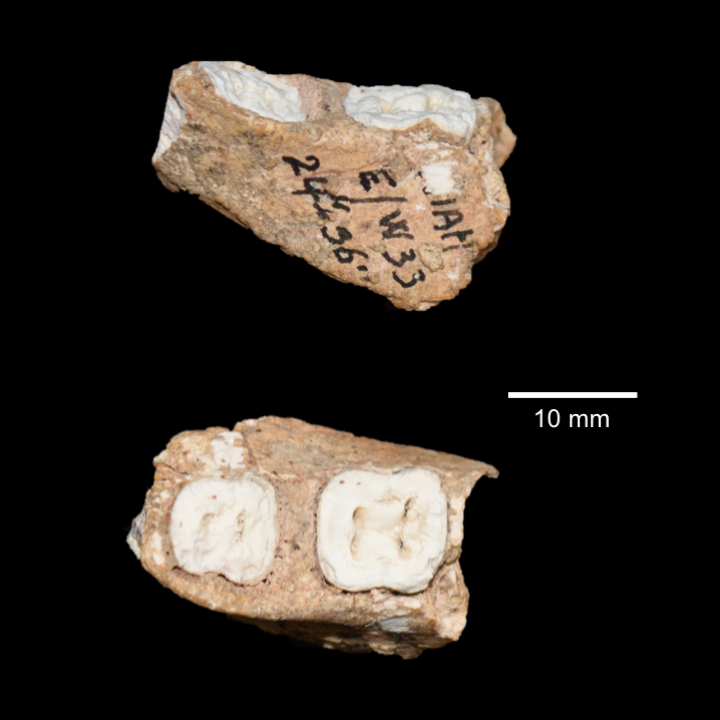
**

**S1 Fig. Mandible E/W 33 24-66" from the West Mouth of the Niah Caves.**

Supplement: S1 Fig — (DOCX) [file pone.0196633.s006.docx]
