# Supplementary material for: Rare Late Pleistocene-early Holocene human mandibles from the Niah Caves (Sarawak, Borneo)
Source: PLoS One. 2018 Jun 6;13(6):e0196633. doi: 10.1371/journal.pone.0196633 (PMC5991356; doi:10.1371/journal.pone.0196633)
Supplement: S2 Fig — (DOCX) [file pone.0196633.s007.docx]

**
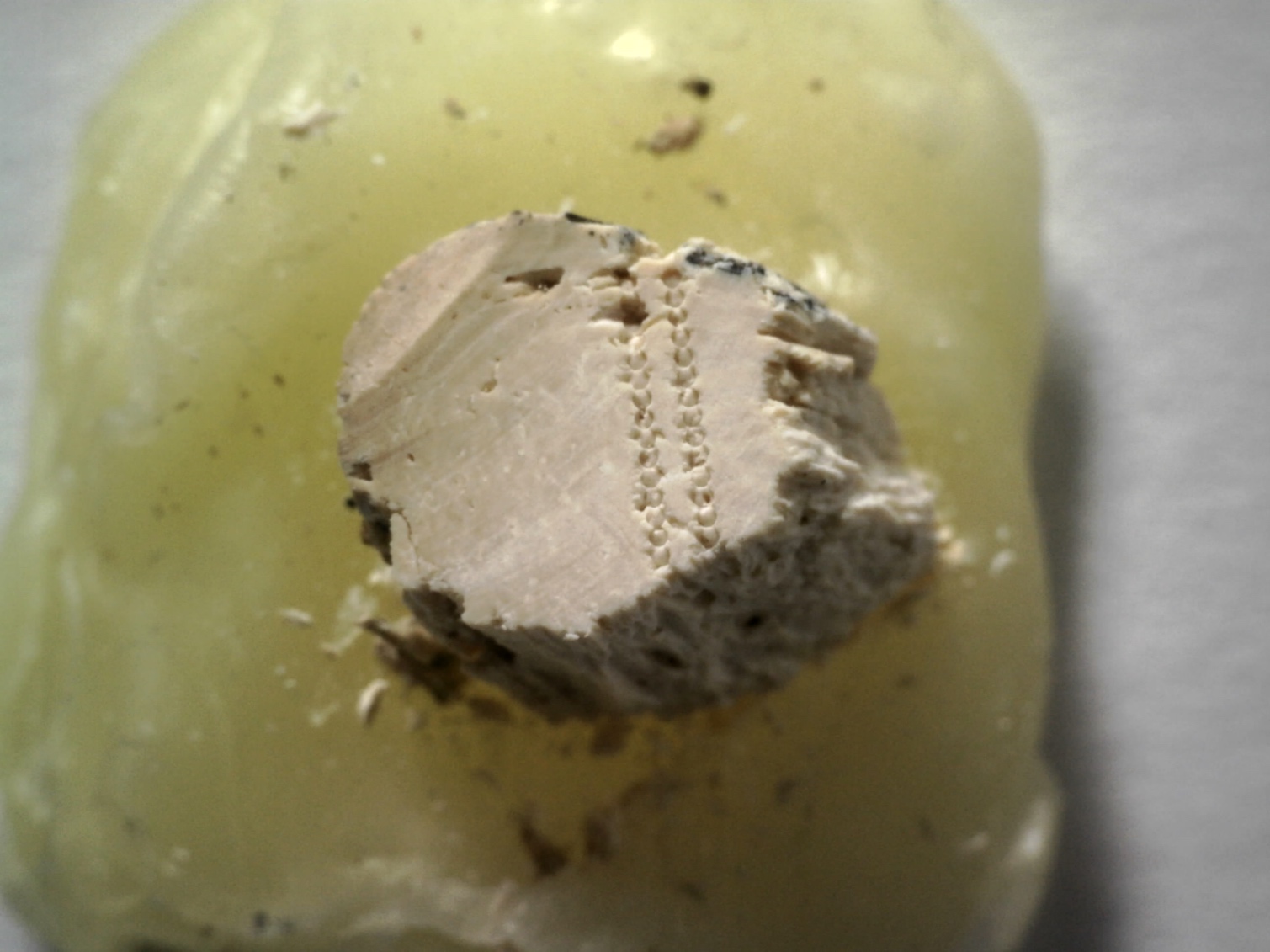
**

**S2 Fig. Fragment of bone from medial surface of E/B1 100" showing the two laser tracks from laser ablation U-series analyses.**
